# A mouse model of non-motor symptoms in Parkinson's disease: focus on pharmacological interventions targeting affective dysfunctions

**DOI:** 10.3389/fnbeh.2014.00290

**Published:** 2014-08-27

**Authors:** Alessandra Bonito-Oliva, Débora Masini, Gilberto Fisone

**Affiliations:** Department of Neuroscience, Karolinska InstitutetStockholm, Sweden

**Keywords:** Parkinson's disease, gait, olfaction, depression, anxiety, dopamine, noradrenaline, mouse

## Abstract

Non-motor symptoms, including psychiatric disorders, are increasingly recognized as a major challenge in the treatment of Parkinson's disease (PD). These ailments, which often appear in the early stage of the disease, affect a large number of patients and are only partly resolved by conventional antiparkinsonian medications, such as L-DOPA. Here, we investigated non-motor symptoms of PD in a mouse model based on bilateral injection of the toxin 6-hydroxydopamine (6-OHDA) in the dorsal striatum. This model presented only subtle gait modifications, which did not affect horizontal motor activity in the open-field test. Bilateral 6-OHDA lesion also impaired olfactory discrimination, in line with the anosmia typically observed in early stage parkinsonism. The effect of 6-OHDA was then examined for mood-related dysfunctions. Lesioned mice showed increased immobility in the forced swim test and tail suspension test, two behavioral paradigms of depression. Moreover, the lesion exerted anxiogenic effects, as shown by reduced time spent in the open arms, in the elevated plus maze test, and by increased thigmotaxis in the open-field test. L-DOPA did not modify depressive- and anxiety-like behaviors, which were instead counteracted by the dopamine D2/D3 receptor agonist, pramipexole. Reboxetine, a noradrenaline reuptake inhibitor, was also able to revert the depressive and anxiogenic effects produced by the lesion with 6-OHDA. Interestingly, pre-treatment with desipramine prior to injection of 6-OHDA, which is commonly used to preserve noradrenaline neurons, did not modify the effect of the lesion on depressive- and anxiety-like behaviors. Thus, in the present model, mood-related conditions are independent of the reduction of noradrenaline caused by 6-OHDA. Based on these findings we propose that the anti-depressive and anxiolytic action of reboxetine is mediated by promoting dopamine transmission through blockade of dopamine uptake from residual noradrenergic terminals.

## Introduction

Parkinson's disease (PD) is classically defined by the progressive degeneration of the dopaminergic nigro-striatal pathway and by the emergence of rigidity, tremor, and bradykinesia (Braak et al., 2003; Jankovic, 2008). However, PD is also accompanied by non-motor symptoms, including olfactory loss, cognitive decline, depression and anxiety, which frequently appear in the early stages or even during the pre-motor phase of the disease (Ward et al., 1983; Aarsland et al., 2004; Chaudhuri and Schapira, 2009). Psychiatric symptoms have been related to monoaminergic deficits within components of the limbic system implicated in emotional and affective functions. In fact, the progressive death of midbrain dopaminergic neurons in PD is paralleled by the concomitant degeneration of noradrenergic and serotonergic systems (Halliday et al., 1990; Braak et al., 2003; Remy et al., 2005; Kish et al., 2008; Frisina et al., 2009).

Although L-DOPA remains the gold standard therapy for the motor symptoms of PD, its efficacy on mood-related dysfunctions is limited and its chronic administration correlates with the emergence of depression and anxiety (Damasio et al., 1971; Marsh and Markham, 1973; Vazquez et al., 1993; Choi et al., 2000; Kim et al., 2009; Negre-Pages et al., 2010; Eskow Jaunarajs et al., 2011). In addition, the use of L-DOPA is complicated by the development of motor side effects such as dyskinesia (Obeso et al., 2000). In this context, drugs acting preferentially on dopamine D2 receptors (D2Rs) and D3 receptors (D3Rs) represent a potential alternative (Hametner et al., 2012). For instance, the D2R/D3R agonist, pramipexole, has been shown to reduce depression and anxiety in clinical trials (Leentjens et al., 2009; Barone et al., 2010).

Common antidepressant agents, such as noradrenaline and serotonin reuptake inhibitors, have also been tested in parkinsonian patients. Tricyclic antidepressants acting on noradrenaline reuptake, such as desipramine and reboxetine, as well as citalopram, a selective serotonin reuptake inhibitor, reduce depression associated to PD (Lemke, 2002; Devos et al., 2008). Nortriptyline, which blocks noradrenaline and serotonin reuptake, is also able to reduce depression in PD patients (Menza et al., 2009). However, mood disorders in PD are still considered difficult to treat, possibly due to the low dosage of appropriate medications normally employed to avoid worsening of motor symptoms (Weintraub et al., 2003). In this regard, the development of reliable preclinical models reproducing the non-motor symptoms of parkinsonian patients is critical to further characterize the mechanisms implicated in these conditions and to test novel therapeutic strategies.

We have previously described a cognitive deficit in a mouse model of PD generated by partial bilateral lesion of the catecholamine system, achieved with intra-striatal injections of 6-hydroxydopamine (6-OHDA). This procedure decreases tyrosine hydroxylase (TH) immunoreactivity, as well as dopamine and noradrenaline levels, in the striatum and hippocampus. These effects are paralleled by a reduction of TH-positive cells in the substantia nigra pars compacta and in the locus coeruleus (Bonito-Oliva et al., 2014). Here, we show that the same experimental model displays a series of anomalies associated to parkinsonism, including gait impairment and olfactory deficit. Notably, this model also reproduces mood dysfunctions analogous to those observed in PD patients. Various drugs interfering with dopaminergic and noradrenergic transmission were tested for their ability to counteract these latter conditions.

## Materials and methods

### Animals

Male C57BL/6J mice (25–30 g; Taconic, Tornbjerg, Denmark) were housed under a 12 h light-dark cycle with food and water *ad libitum*. Experiments were carried out during the light phase, in accordance with the guidelines of Research Ethics Committee of Karolinska Institutet, Swedish Animal Welfare Agency and European Communities Council Directive 86/609/EEC.

### Drugs

The following drugs were used: benserazide hydrochloride, 3,4-dihydroxy-l-phenylalanine (L-DOPA), 6-hydroxydopamine hydrochloride (6-OHDA) and desipramine hydrochloride (Sigma-Aldrich, St. Louis, MO, USA), pramipexole dihydrochloride (Tocris, Bristol, UK) and reboxetine mesylate (Abcam Biochemicals, Cambridge, UK). 6-OHDA was dissolved in saline containing 0.02% ascorbic acid and locally injected in the brain. Desipramine (25 mg/kg, i.p.), pramipexole (0.6 mg/kg, s.c.), reboxetine (20 mg/kg, i.p.) and L-DOPA [10 mg/kg, i.p.; administered together with the peripheral DOPA decarboxylase inhibitor, benserazide (7.5 mg/kg)] were dissolved in saline. All drugs were injected in a volume of 10 ml/kg. Desipramine was administered 30 min before the injection of 6-OHDA. The other drugs were administered for four consecutive days preceding the experiment. The animals were tested 30 min after the last drug administration.

### 6-OHDA lesion

Mice were anesthetized with a mixture of Hypnorm® (VetaPharma Ltd, Leed, UK), midazolam (5 mg/ml) (Hameln Pharmaceuticals GmbH, Hameln, Germany) and water (1:1:2 in a volume of 10 ml/kg) and mounted in a stereotaxic frame (David Kopf Instruments, Tujunga, CA, USA). Each mouse received a bilateral injection of 1 μl of 6-OHDA (4 μg/μl) into the dorsal-lateral striatum, according to the following coordinates (mm) (Franklin and Paxinos, 1997): antero-posterior +0.6, medio-lateral ±2.2, and dorso-ventral −3.2. Control sham-lesioned (sham) mice were injected with the same volume of vehicle (0.9% saline and 0.02% ascorbic acid). After surgery, the animals were allowed to recover for 3 weeks.

### Experimental plan

A total of 154 sham and 6-OHDA-lesioned mice were randomly assigned to the different experimental groups and examined in batteries of behavioral tests, which were performed sequentially according to their increasing invasiveness (i.e., gait analysis, open field and tail suspension; or odor discrimination, elevated plus maze and forced swim test). Each test was separated by a resting period of 7–10 days. Additional groups of sham and 6-OHDA lesioned mice were used to evaluate the effects of drugs (i.e., L-DOPA, pramipexole, desipramine and reboxetine) in the elevated plus maze and forced swim test. Four percent of the animals initially involved in the study died during the post-operatory phase, and 5% of the animals injected with 6-OHDA were excluded from the behavioral analysis due to insufficient depletion of striatal TH (see Results 3.1).

### Behavioral analyses

#### Gait analysis

The pattern of motor coordination of sham and 6-OHDA-lesioned mice was examined using ventral plane videography (Amende et al., 2005; Hampton and Amende, 2010). The apparatus consisted of a motor-driven transparent treadmill belt (Exer Gait XL, Columbus Instruments, USA) and a high-speed digital video camera (100 fps) to record the ventral view of the treadmill belt, as reflected by an angled mirror. An adjustable compartment (17×5 cm) was mounted over the treadmill belt to maintain the mouse in the view of the camera, limit lateral movements and reduce parallax errors. All tested mice had similar body lengths (mean snout-vent size 8 ± 0.6 cm) and were first habituated to a still treadmill for 3 min, followed by 1 min during which the speed was progressively increased from 0 to 17 cm/s (Heglund and Taylor, 1988; Leblond et al., 2003). Once the testing speed was achieved gait was recorded during three 20 s trials separated by 1 min inter-trials periods. The contacts made with the treadmill by each individual paw were determined using the TreadScan software (Treadscan 4.0, Clever Sys, Inc., Reston, VA, USA). For each animal at least 20 steps/paw were analyzed. The following standard gait parameters were measured: stride duration (time separating two consecutive contacts with the treadmill made with the same paw), stance time (portion of the stride during which the paw is in contact with the treadmill) and swing time (portion of the stride during which the paw is not in contact with the treadmill). All measurements were averaged. The time during which the animals ran to the front, drifted backward on the belt, or explored the chamber were excluded from the analysis.

#### Olfactory analysis

Olfaction was measured using a modified version of the olfactory habituation/dishabituation test, as previously described (Wrenn et al., 2003; Crawley et al., 2007; Stack et al., 2008; Yang and Crawley, 2009). On the test day, each mouse was habituated for 2 h to a bedding-supplied home cage (30 × 15 × 15 cm). Next, a dry cotton tipped swab was placed in the cage for 30 min. At the end of this habituation phase, the animal was exposed to a sequence of five odors delivered through cotton swabs, according to the following sequence: water, almond flavor, citrus flavor, social odor 1(*social*_1_), social odor 2(*social*_2_). The almond and citrus odors were prepared by diluting almond and citrus flavors (Dr. Oetker) in distilled water (1:600); each social odor was obtained by pooling the urine of two adult male mice. Each odor was presented for three consecutive 2 min periods, separated by approximately 30 s inter-trials. For every exposure, the cotton swab was freshly prepared with a fixed amount of solution (5 and 4 μl for non-social and social odors, respectively). The test was video-recorded and exploratory activity (time during which the mouse nose was in contact with the cotton swab, or directed toward it at a distance ≤2 cm) was measured by an observer blind to the experimental groups. This protocol allows to determine the *habituation* to a specific odor, indicated by the progressive decreased in exploration time for the same odor during the three presentations, and the *dishabituation*, measured as increased exploration time for a new odor, which is an index of the ability to discriminate olfactory novelty (Silverman et al., 2011).

#### Forced swim test

In the forced swim test, each mouse was placed in a glass cylinder (25 cm in height and 13 cm in diameter), filled up to 15 cm with tap water at a temperature of 22°C and let swim for 10 min. At the end of the test, the mouse was removed from the cylinder, gently dried and placed in the home cage under warm light, for 10 min. The swimming behavior of each animal was video-recorded and analyzed by an observer blind to the experimental groups. The immobility time (defined as the time spent by the mouse floating, with only minimal movements to keep the head above the water surface) was measured and considered as an index of depression (Castagne et al., 2009).

#### Tail suspension test

In the tail suspension test, mice were suspended by the tail, with adhesive tape, on a metal rod installed 30 cm above the plane of a laboratory bench and left in this position for 6 min. The behavior of each mouse was video-recorded and analyzed by an observer blind to the experimental groups. The immobility time (defined as the time during which the animal was hanging passively and motionless) was measured and considered as an index of depression (Steru et al., 1985).

#### Elevated plus maze test

The elevated plus maze apparatus was composed of four black plastic arms, arranged as a cross, located 55 cm above the plane of a laboratory bench and illuminated by a 60 w lamp located above the apparatus. Two close arms, opposite to each other were enclosed by lateral walls (50 × 6 × 40 cm), whereas the other two open arms were without walls (50 × 6 × 0.75 cm); the close and open arms delimited a small squared area (6 × 6 cm) called center. Each mouse was placed into the center of the maze, facing one of the two open arms, and its behavior was video-recorded for 5 min. The time spent by the mice in each of the three compartments (open, close, center) was measured by an observer blind to the experimental groups. The propensity to avoid the open arms is considered as an index of anxiety (Lister, 1987).

#### Open field test and thigmotaxis

Thigmotaxis was determined in an open field box (40 × 40 × 40 cm), virtually divided in a peripheral and a central zone of equivalent area. Each mouse was allowed to explore the apparatus for 15 min and its behavior was recorded by a video camera connected to an automated tracking system (BIOBSERVE GmbH, Germany). In this test, the preferential exploration of the peripheral zone of the open field is considered an index of anxiety (Simon et al., 1994).

### Western blotting

At the end of each test battery, mice were sacrificed by decapitation, left and right striata were dissected out, sonicated in 750 μl of 1% SDS and boiled for 10 min. TH-immunoreactivity was determined by Western blotting as previously described (Bonito-Oliva et al., 2014). Briefly, aliquots (5 μl) from sonicated striata were used for protein quantification with the BCA assay kit (Pierce, Rockford, IL, USA). Equal amounts of protein (5 μg) for each sample were loaded onto 10% polyacrylamide gels. Proteins were separated by SDS-PAGE and transferred overnight to polyvinylidene difluoride membranes (GE Healthcare, Little Chalfont, UK). The membranes were immunoblotted using a mouse anti-TH antibody (1:3000, Chemicon International, Temecula, CA, USA), and then incubated in horseradish peroxidase-conjugated secondary anti-mouse antibody (1:30000). The protein signal was visualized by ECL (Pierce, Rockford, IL, USA) and quantified using Quantity One software (Bio-Rad). The levels of TH were expressed as percent of those in sham striatum and used to assess the effectiveness of the lesion.

### Statistical analysis

Data were analyzed using One-Way ANOVA and *post-hoc* comparisons between groups were made using the Fisher's or Sidak-Bonferroni test. Student's *t*-test with equal variances was used to analyze experiments with two groups. For the olfactory habituation/dishabituation tests, data were analyzed using repeated measures ANOVA followed by Newman-Keuls *post-hoc* comparison test.

## Results

### Effect of partial 6-OHDA lesion on striatal TH

We have previously shown that the protocol of 6-OHDA lesion utilized in this study produces a partial decrease in TH immunoreactivity in the striatum (Bonito-Oliva et al., 2014). In line with this result, Western blotting quantification showed a reduction of striatal TH immunoreactivity ranging from 63 to 74%. The animals that did not match this criterion (5% of the mice injected with 6-OHDA) were excluded from the behavioral analysis.

### Partial bilateral 6-OHDA lesion disrupts step cycle regularity by reducing hindlimb stance

In line with previous work indicating the limited effect of the partial 6-OHDA lesion on motor function (Bonito-Oliva et al., 2014), lesioned mice did not differ from sham mice with regard to stride length (distance between two successive footfalls of the same paw), stride frequency and stride duration (time lag between two successive touches down of the reference limb) (Table 1). Moreover, alignment of the body axis to the direction of the movement, body swing, and track width (i.e., measure of the distance between the two front or hind paws) were similar in 6-OHDA-lesioned and sham mice (Table 1).

**Table 1:** **Summary of gait parameters in sham and 6-OHDA-lesioned mice**.

	**Sham**	**6-OHDA-lesioned**
Front track width (mm)	10.65±0.63	10.24±0.83
Hind track width (mm)	18.85±1.02	19.22±1.45
Stride length (mm)	46.35±1.11	50.08±1.23
Normal stride frequency (Hz)	3.67±0.07	3.52±0.05
Stride duration (ms)	223.0±7.1	232.5±6.3
% Forelimb swing time	45.96±1.44	47.70±1.42
% Hindlimb swing time	44.41±1.57	51.70±2.08^*^

*Twenty-two sham mice and 17 6-OHDA-lesioned mice were examined on a treadmill set at a speed of 17 cm/s. For each animal, data were collected from 20 to 25 strides and averaged. Data are expressed as mean ± s.e.m*.

* *p < 0.05 vs. Sham group, Student's t-test*.

**Figure 1:**
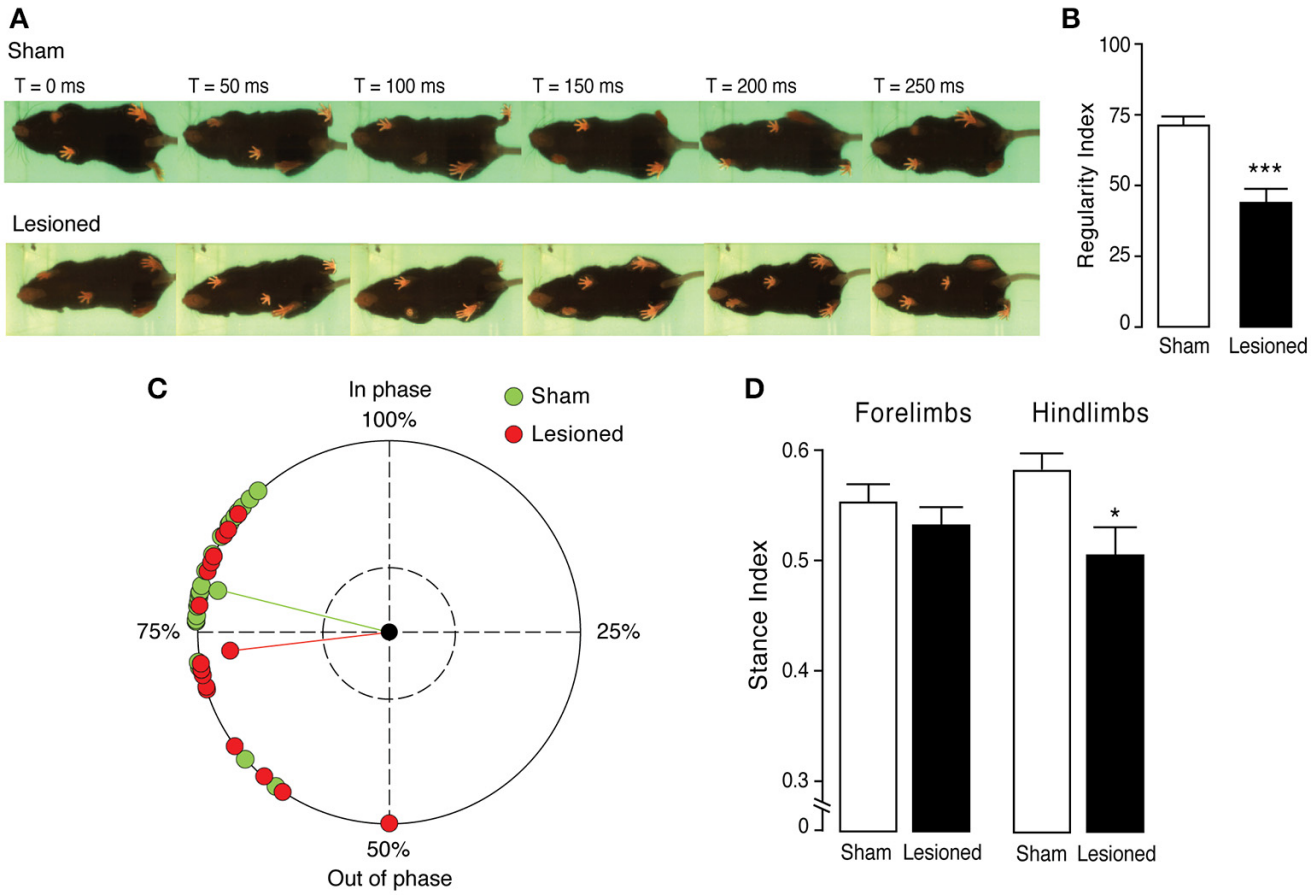
**Disruption of step cycle regularity in 6-OHDA-lesioned mice**. Gait dynamics and pattern of motor coordination during locomotion were analyzed in sham (*n* = 22) and 6-OHDA-lesioned (Lesioned) (*n* = 17) mice. **(A)** Representative images of a complete step cycle (250 ms), shown in six consecutive frames taken every 50 ms, performed by a sham (upper) and a 6-OHDA-lesioned (bottom) mouse. **(B)** Bar graph showing the regularity index (expressed as percentage of correct step sequences over total number of step cycles) in sham and 6-OHDA-lesioned mice. **(C)** The coupling interval (expressed in percent) of diagonal limbs for each sham and 6-OHDA-lesioned mouse is represented in a circular graph, which shows in phase or out of phase touches performed on the treadmill. Individual circles represent the average phase value of each animal. The dashed inner circle represents the cut off of significance. The length of the green and red lines is proportional to the statistical significance and the two circles connected to the lines represent the mean phase value of sham (green) and 6-OHDA-lesioned (red) mice. **(D)** Bar graph showing the relative contribution to the stride (expressed as percentage) of forelimb stance (left) and hindlimb stance (right), in sham and 6-OHDA-lesioned mice. Data are expressed as mean ± s.e.m. ^***^p < 0.0001, ^*^p < 0.01 vs. sham mice, Student's *t*-test.

6-OHDA-lesioned mice were then analyzed for step cycle regularity, which indicates the frequency of correct step cycles (i.e., fully coordinated motion in which each paw is placed on time every four times) during movement (Redondo-Castro et al., 2013). Sham and 6-OHDA-lesioned mice performed the same number of step cycles during the test (98 ± 9 and 80 ± 7 step cycles, respectively). However, as shown in the ventral videogram (Figure 1A), lesioned mice differ from sham mice with regard to the way in which they bring their paws in contact with the ground, which results in a significant impairment in step-cycle regularity (*p* < 0.0001, Student's *t*-test) (Figure 1B).

Mice were further analyzed for inter-limb coordination by examining coupling of the homolateral limbs (i.e., limbs on the right or left side), coupling of the homologous limbs (i.e., forelimbs or hindlimbs) and coupling of the diagonal limbs (i.e., right forelimb with left hindlimb and vice versa). Whereas no differences were observed in the first two parameters (data not shown), diagonal coupling was out of phase in lesioned mice. Sham mice showed a coupling interval (i.e., percent of time during which the diagonal limbs contact the ground at the same time) over 75% (78.6 ± 1.4%), which is in line with what has been previously described in C57BL/6 mice (Leblond et al., 2003) (Figure 1C). This parameter was reduced to 72.7 ± 2.4% in lesioned mice (*p* < 0.05, Student's *t*-test) (Figure 1C). In line with these gait abnormalities, stride cycle analysis of 6-OHDA-lesioned mice revealed increased hindlimb swing time (cf. Table 1) and shorter stance (time with the limb in contact with the ground), affecting selectively the hindlimbs (*p* < 0.01, Student's *t*-test) (Figure 1D). Note that for all the above-mentioned measures, no differences were found for gait variability (standard deviation) and coefficient of variability (calculated as 100 × standard deviation/mean value).

### Reduced olfactory discrimination in 6-OHDA-lesioned mice

**Figure 2:**
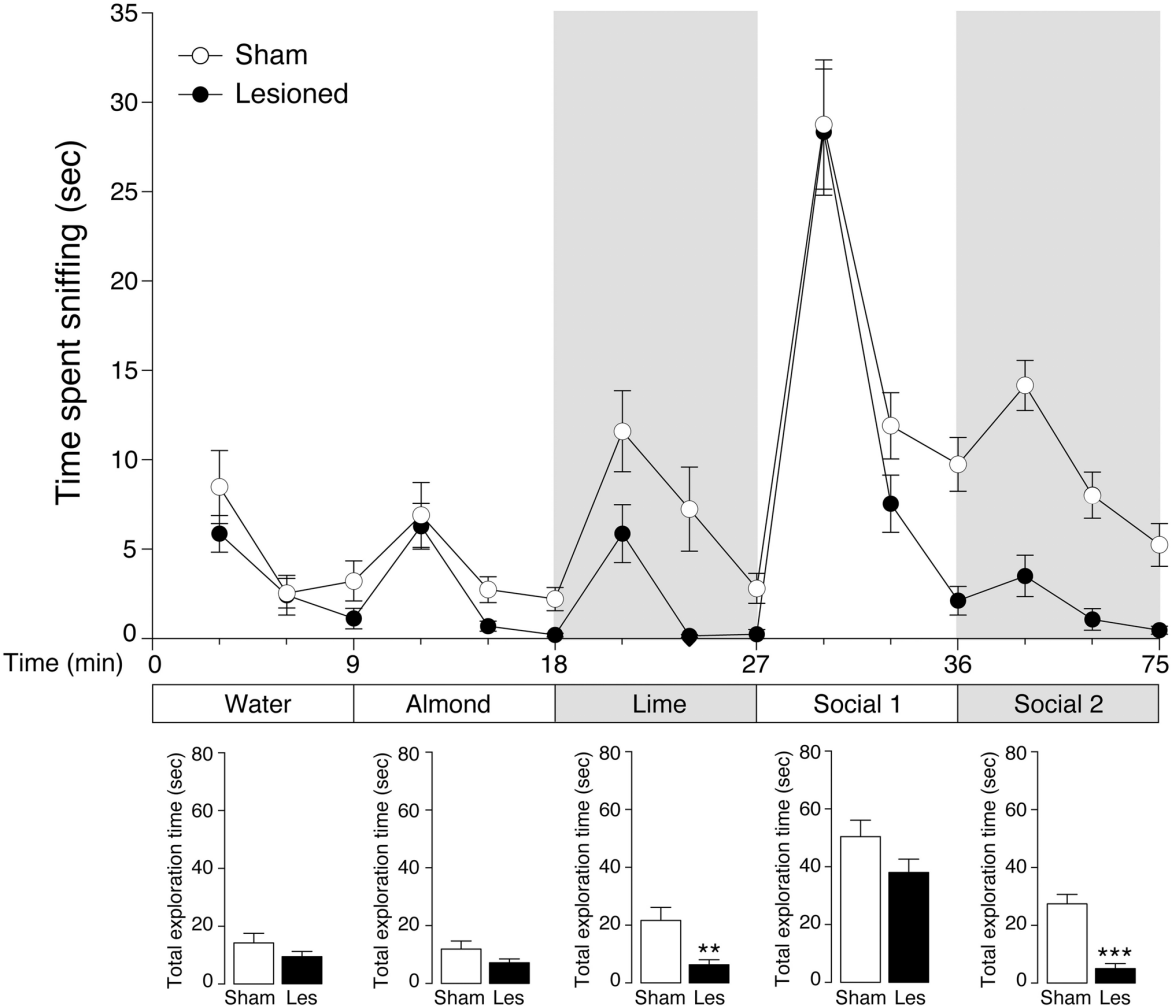
**Reduced olfactory discrimination in 6-OHDA-lesioned mice**. The ability of sham (*n* = 9) and 6-OHDA-lesioned (Lesioned) (*n* = 12) mice to recognize different odors was examined in the habituation/dishabituation test. Exploratory activity was measured as time (s) spent by the animals sniffing water- (control), or odor-wet cotton swabs. The mice were exposed to three consecutive 2 min presentations of each odor, according to the following order: water, non-social (almond, lime) and social (*social*_1_ and *social*_2_), with 30 s intervals between each exposure. Lower panels show the total exploration time (s) spent by sham and 6-OHDA-lesioned mice sniffing individual odors (water, almond, lime, *social*_1_, *social*_2_). The gray background indicates the second odor presented for each category (non-social and social). Data are presented as mean ± s.e.m. ^**^p < 0.01, ^***^p < 0.0001 vs. sham mice, Student's *t*-test.

Loss of olfaction (anosmia) is among the earliest manifestations of PD. This disturbance affects most patients and typically precedes the motor symptoms of the disease (Ward et al., 1983; Doty et al., 1988; Braak and Del Tredici, 2009). Anosmia was examined by comparing the olfactory discriminative ability of mice with partial 6-OHDA lesion with that of sham mice. Specifically, we measured the progressive decrease in exploration during repeated presentations of the same odor (*habituation*), as well as the tendency to explore for a longer period of time a new odor (*dishabituation*) (Woodley and Baum, 2003). Previous work showed that the 6-OHDA lesion utilized in this study does not affect exploratory activity (Bonito-Oliva et al., 2014). In line with this observation, lesioned mice showed normal baseline exploratory activity for the neutral stimulus *water* (Figure 2). Therefore, in this test, the exploratory activity was considered an index of attractiveness to the odor itself (Macknin et al., 2004; Crawley et al., 2007).

Sham and 6-OHDA-lesioned mice showed olfactory habituation for all odors tested, as indicated by the decreases in exploration time displayed by the two groups of mice during the 3 consecutive presentations of the same odor (Figure 2, upper panel). One-Way ANOVA with repeated measure (trial) indicated a progressive reduction in exploration time for each odor in sham [*water F*_(2, 5)_ = 6.503, *p* < 0.05; *almond F*_(2, 7)_ = 4.643, *p* < 0.05; *lime F*_(2, 7)_ = 11.015, *p* < 0.01; *social*_1_ *F*_(2, 7)_ = 13.957, *p* < 0.01; *social*_2_ *F*_(2, 7)_ = 19.499, *p* < 0.01] and in 6-OHDA-lesioned mice [water *F*_(2, 10)_ = 7.893, *p* < 0.01; *almond F*_(2, 10)_ = 11.025, *p* < 0.01; *lime F*_(2, 10)_ = 6.009, *p* < 0.05; *social*_1_ *F*_(2, 10)_ = 32.245, *p* < 0.0001; *social*_2_ *F*_(2, 10)_ = 4.181, *p* < 0.05]. We also analyzed the effect of the 6-OHDA lesion on the habituation profile for each odor. Two-way ANOVA with repeated measure (trial) revealed a significant lesion effect for *lime* [*F*_(1, 19)_ = 12.482, *p* < 0.01] and for *social*_2_ [F_(1, 19)_ = 42.061, *p* < 0.0001] and a significant lesion × trial interaction in the case of odor *social*_2_ [F_(2, 38)_ = 8.278, *p* < 0.01; *p* < 0.0001 for all trials, Newman-Keuls *post-hoc*].

The ability of the mice to detect olfactory novelty (*dishabituation*) was evaluated based on the increase in time spent exploring the first swab of a new odor (Figure 2, upper panel). For each odor replacement, data were analyzed by One-Way ANOVA with repeated measure (trial) in sham [*water* to *almond F*_(1, 6)_ = 6.174, *p* < 0.05; *almond* to *lime F*_(1, 8)_ = 20.420, *p* < 0.01, *lime* to *social*_1_ *F*_(1, 8)_ = 64.442, *p* < 0.0001; *social*_1_ to *social*_2_ *F*_(1, 8)_ = 11.986, *p* < 0.01] and 6-OHDA-lesioned mice [*water* to *almond F*_(1, 11)_ = 12.896, *p* < 0.01, *almond* to *lime F*_(1, 11)_ = 12.397, *p* < 0.01, lime to *social*_1_ *F*_(1, 11)_ = 61.941, *p* < 0.0001, social_1_ to *social*_2_ not significant] (Figure 2). We then analyzed the effect of the 6-OHDA lesion on the dishabituation for each odor replacement by Two-Way ANOVA with repeated measure (trial). We found a significant lesion × trial interaction when the mice were exposed to a new odor within the same category non-social: *almond* to *lime* [*F*_(1, 19)_ = 6.949, *p* < 0.01] and social: *social*_1_ to *social*_2_ [*F*_(1, 19)_ = 39.65, *p* < 0.0001]. Notably, no interaction effect was found when the animals were exposed for the first time to a non-social (*almond*), or social (*social*_1_) odor.

Finally, we analyzed the total time spent by the animals exploring the three consecutive presentations of the same odor (Figure 2, lower panels). Sham and 6-OHDA-lesioned mice displayed similar exploration time for *water*, *almond* and *social*_1_. In contrast, 6-OHDA-lesioned mice showed decreased exploration time for *lime* and *social*_2_ (*p* < 0.01 and *p* < 0.0001 vs. sham-lesioned mice, respectively; Student's *t*-test).

Altogether, these results indicated that the partial 6-OHDA lesion causes an olfactory deficit, as indicated by the reduced response of lesioned mice in the habituation/dishabituation test, when presented with odor stimuli that belong to the same category.

### Bilateral 6-OHDA lesion induces depression- and anxiety-like behaviors in the mouse

**Figure 3:**
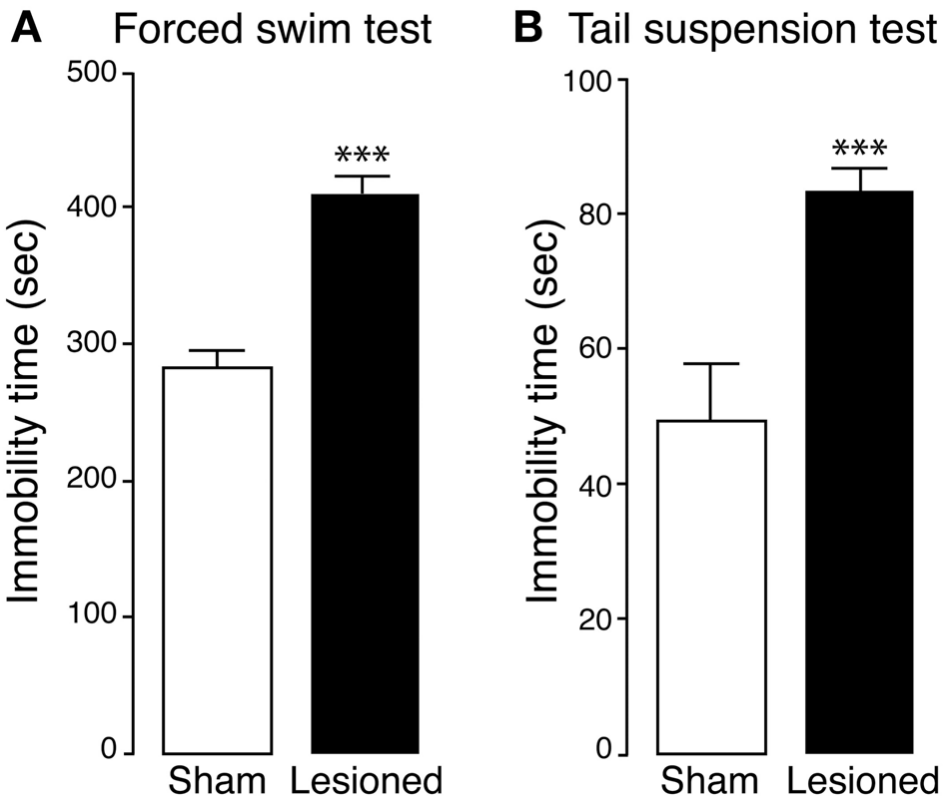
**Depression-like phenotype in 6-OHDA-lesioned mice**. Sham and 6-OHDA-lesioned (Lesioned) mice were tested for depression-like behavior in the forced swim test **(A)** and tail suspension test **(B)**. Bar graphs show the immobility time (s) of sham and 6-OHDA-lesioned mice, during the forced swim test (**A**; test duration, 10 min, sham *n* = 12, lesioned *n* = 15) and the tail suspension test (**B**; test duration 6 min, sham *n* = 15, lesioned *n* = 12). Data are expressed as mean ± s.e.m. ^***^p < 0.0001 vs. sham mice, Student's *t*-test.

The effect produced by partial 6-OHDA lesion on depressive-like behavior was examined in the forced swim and tail suspension tests (Castagne et al., 2009; Ginsberg et al., 2012). We found that, in the forced swim test, 6-OHDA-lesioned mice displayed a longer immobility time (considered an index of depression) in comparison to sham mice (*p* < 0.0001, Student's *t*-test) (Figure 3A). A similar difference was observed when sham and 6-OHDA-lesioned mice were examined in the tail suspension test (*p* < 0.0001, Student's *t*-test) (Figure 3B).

**Figure 4:**
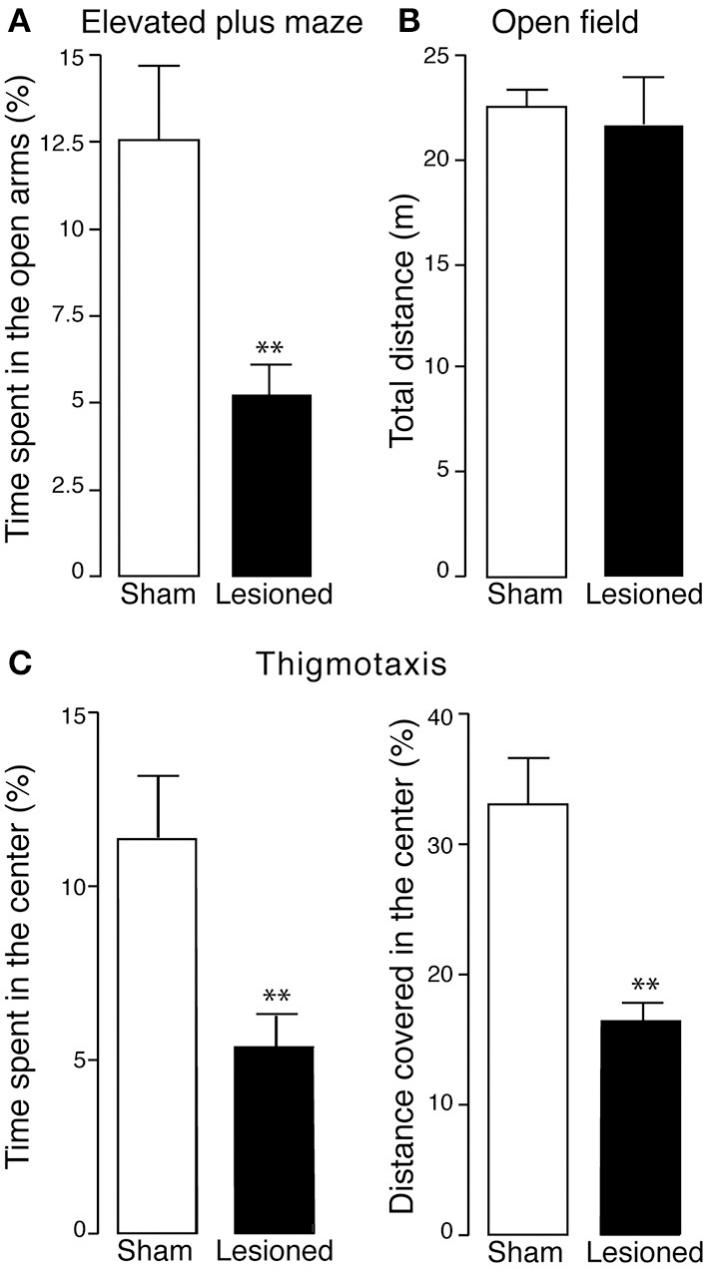
**Anxiety-like phenotype in 6-OHDA-lesioned mice**. Sham and 6-OHDA-lesioned (Lesioned) mice were tested for anxiety-like behavior in the elevated plus maze **(A)** and open field **(B,C)**. **(A)** In the elevated plus maze, the time spent in the open arms (% of the test duration) by sham (*n* = 12) and 6-OHDA-lesioned mice (*n* = 15) mice, was measured during a 5 min test. **(B)** In the open field, the total distance (m) covered by sham (*n* = 11) and 6-OHDA-lesioned mice (*n* = 8) mice was measured, during a 15 min test. **(C)** Thigmotaxis was evaluated in the open field by measuring the time spent (% of total test duration) and the distance covered (% of total distance covered) in the center of the apparatus (left and right panel, respectively). Data are expressed as mean ± s.e.m. ^**^p < 0.01 vs. sham mice, Student's *t*-test.

Mice were then examined for anxiety-like behavior in the elevated plus maze. During the 5 min test, sham and 6-OHDA-lesioned mice spent the same amount of time in the center of the apparatus and showed a clear preference for the close arms over the open arms (data not shown). However, this preference was more pronounced in the lesioned mice, which spent significantly less time in the open arms, as compared to sham mice (*p* < 0.01, Student's *t*-test) (Figure 4A).

The anxiety-like phenotype induced by the lesion was confirmed by testing the mice in the open field (Figures 4B,C). In line with previous work, lesion with 6-OHDA did not affect horizontal motor activity, as indicated by similar distances covered by sham and 6-OHDA-lesioned mice during the test (15 min) (Figure 4B). However, 6-OHDA-lesioned mice showed a pronounced increase in thigmotaxis when compared to sham mice, as indicated by less time spent in the center of the open field (*p* < 0.01, Student's *t*-test) and shorter distance covered in the center of the open field (*p* < 0.01, Student's *t*-test) (Figure 4C). Taken together these data demonstrate that, in the mouse, a partial catecholamine lesion induced by 6-OHDA leads to behaviors indicative of affective dysfunctions.

### Depression- and anxiety-like behaviors induced by bilateral 6-OHDA lesion are reverted by pramipexole, but not by L-DOPA

**Figure 5:**
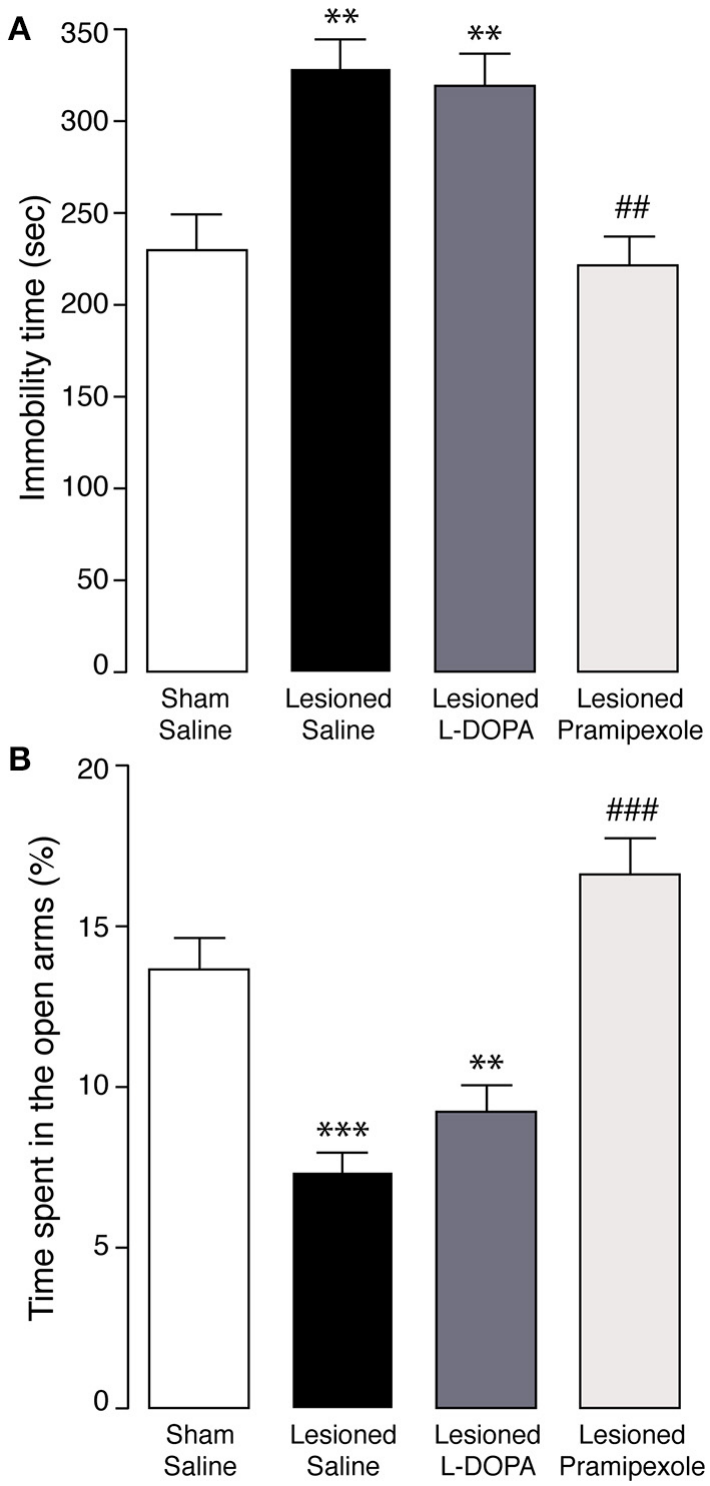
**Effect of L-DOPA and pramipexole on depression- and anxiety-like behaviors in 6-OHDA-lesioned mice**. Sham, and 6-OHDA-lesioned mice treated for 4 days with saline, L-DOPA (10 mg/kg), or pramipexole (0.6 mg/kg) were tested in **(A)** the forced swim test, or **(B)** the elevated plus maze test. Bar graphs show the immobility time (s) during the forced swim test (**A**; test duration, 10 min) and the time spent in the open arms (% of the test duration) during the elevated plus maze test (**B**; test duration 5 min). Data are expressed as mean ± s.e.m. (*n* = 4 − 7). ^**^p < 0.01, ^***^p < 0.0001 vs. sham mice, ^##^*p* < 0.01, ^###^*p* < 0.0001 vs. 6-OHDA-lesioned mice treated with saline, One-Way ANOVA, followed by Fisher *post-hoc*.

We next examined the ability of L-DOPA and pramipexole, two drugs commonly used to treat the motor symptoms of PD, to revert the depression- and anxiety-like behaviors produced by bilateral 6-OHDA lesion. In the forced swim test, four consecutive days of treatment with L-DOPA (10 mg/kg) did not reduce the immobility time produced by the lesion (Figure 5A). In contrast, a similar treatment with pramipexole (0.6 mg/kg), a D2R/D3R agonist, reverted the depressive phenotype (Figure 5A). One-Way ANOVA indicated significant treatment effect [*F*_(3, 28)_ = 10.258, *p* < 0.0001] and Fisher's *post-hoc* comparison indicated a significant difference (*p* < 0.01) between 6-OHDA-lesioned mice treated with saline, or pramipexole.

Similar results were obtained when the mice were tested in the elevated plus maze. The animals did not show any treatment-dependent difference in the time spent in the center of the apparatus (data not shown). Importantly, L-DOPA did not counteract the reduction in the time spent in the open arms caused by the lesion with 6-OHDA (Figure 5B). Conversely, following administration of pramipexole the performance of lesioned mice was undistinguishable from that of sham mice, (Figure 5B). One-Way ANOVA indicated a significant effect of the treatment [*F*_(3, 23)_ = 23.286, *p* < 0.0001] and Fisher's *post-hoc* comparison indicated a significant difference (*p* < 0.0001) between 6-OHDA-lesioned mice treated with saline, or pramipexole.

### Depression- and anxiety-like behaviors induced by bilateral 6-OHDA lesion are corrected by reboxetine, but independent of noradrenaline depletion

**Figure 6:**
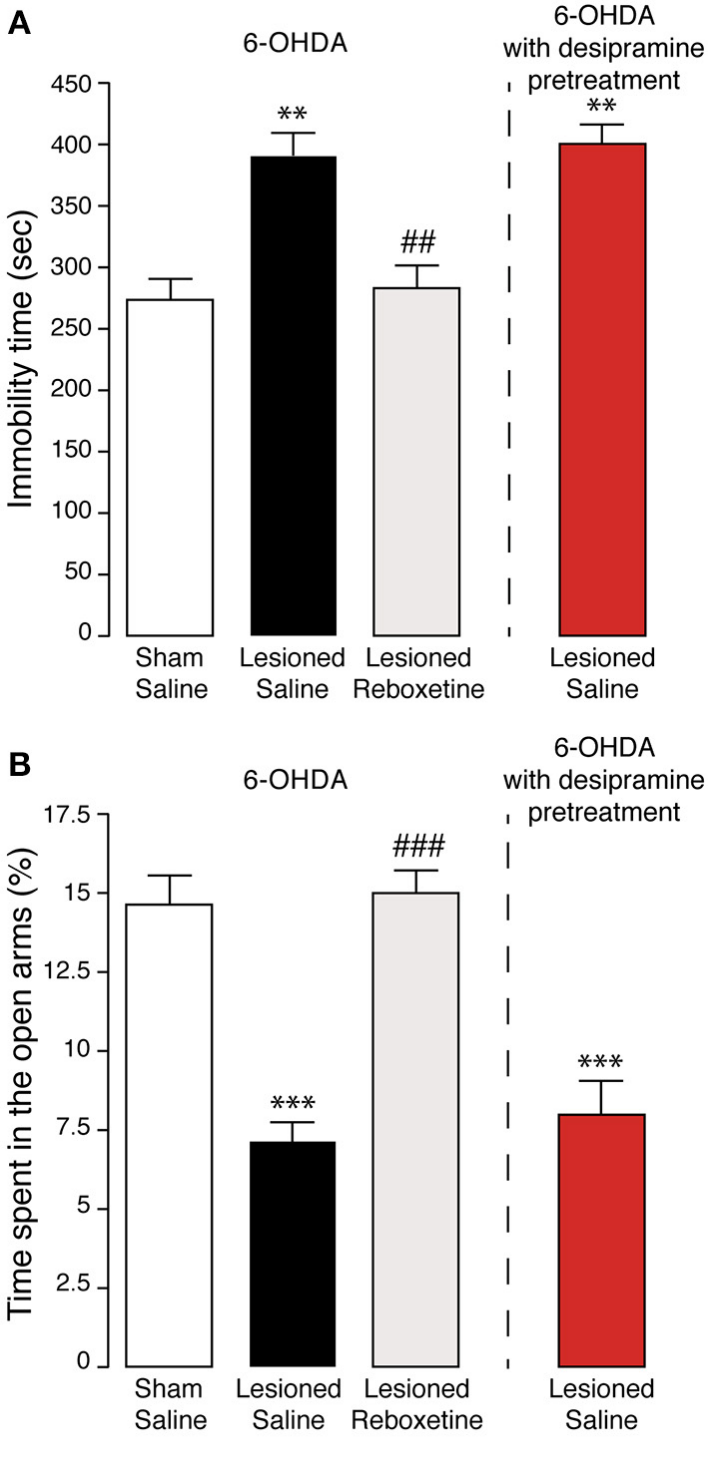
**Effect of noradrenaline reuptake inhibitors on anxiety- and depression-like behaviors induced by 6-OHDA**. Sham, and 6-OHDA-lesioned mice treated for 4 days with saline, or reboxetine (20 mg/kg). An additional group of mice (red) was pretreated with desipramine (25 mg/kg) prior to lesion with 6-OHDA. Bar graphs show the immobility time (s) during the forced swim test (**A**; test duration, 10 min) and the time spent in the open arms (% of the test duration) during the elevated plus maze test (**B**; test duration 5 min). Data are expressed as mean ± s.e.m. (*n* = 7 − 21). ^**^p < 0.01, ^***^p < 0.0001, vs. sham mice, ^##^*p* < 0.01, ^###^*p* < 0.0001 vs. 6-OHDA-lesioned mice treated with saline, One-Way ANOVA, followed by Fisher *post-hoc*.

We have previously shown that the lesion utilized in this study induces a partial depletion of noradrenaline in striatum and hippocampus (Bonito-Oliva et al., 2014). Therefore, we examined the effect produced by the selective noradrenaline reuptake inhibitor, reboxetine. We found that four consecutive days of treatment with reboxetine (20 mg/kg) reverted the effects of the lesion on both forced swim test (Figure 6A) and elevated plus maze (Figure 6B). One-Way ANOVA indicated a significant effect of the treatment [*F*_(3, 43)_ = 14.439, *p* < 0.0001 for the forced swim test and *F*_(3, 43)_ = 16.204, *p* < 0.0001 for elevated plus maze]. Fisher's *post-hoc* comparison indicated a significant difference for both tests between 6-OHDA-lesioned mice treated with saline, or reboxetine (*p* < 0.01 and *p* < 0.0001, respectively).

We further investigated the involvement of the noradrenergic innervation by pre-treating the mice with desipramine, another selective noradrenaline reuptake inhibitor, prior to injection of 6-OHDA. This procedure preserves the noradrenergic neurons, thereby limiting the lesion to the dopaminergic innervation (Bonito-Oliva et al., 2014). Administration of desipramine (25 mg/kg), 30 min prior to 6-OHDA, did not affect the ability of the lesion to increase the immobility time in the forced swim test (Figure 6A), or to reduce the time spent in the open arms, in the elevated plus maze test (Figure 6B). This suggested that the depletion of dopamine caused by 6-OHDA is sufficient to induce affective-like symptoms.

## Discussion

There is ample evidence that PD is manifested not only as a motor disorder, but also as a condition characterized by a variety of non-motor symptoms, ranging from sleep disturbances and anosmia, to cognitive and psychiatric disorders. In this study we show that a partial bilateral lesion of the dorsal striatum with 6-OHDA results in a subtle impairment of gait dynamics, accompanied by olfactory deficit, depression- and anxiety-like behavior. The mild motor impairment indicates that this model reproduces a relatively early stage of PD, which is in line with the partial reduction (70%) in dopamine and noradrenaline previously reported (Bonito-Oliva et al., 2014).

Lesioned mice do not display any reduction in horizontal motor activity and show normal stride length and frequency. These results contrast with previous work in the 1-methyl-4-phenyl-1,2,3,6-tetrahydropyridine (MPTP) mouse model of PD, showing shorter stride length and increased stride frequency (Fernagut et al., 2002; Amende et al., 2005), indicative of a more pronounced motor impairment. However, a detailed analysis of gait dynamics indicates that the lesion with 6-OHDA employed in this study disrupts step coordination and that this impairment is caused by reduced stance affecting the hindlimbs. This gait deficit is in line with the variations in the stance phase observed in PD patients (Ivkovic and Kurz, 2011) and may also be involved in the reduced ability to stand on hindlimbs (rearing), previously observed in the same mouse model (Bonito-Oliva et al., 2014).

Olfactory dysfunction is an early clinical sign in several neurodegenerative disorders. In PD, the incidence of this symptom, manifested as impairment in odor identification, threshold detection, and odor recognition memory, may even surpass that of tremor (Doty et al., 1988; Mesholam et al., 1998). The onset of olfactory deficit precedes the motor symptoms by several years and is regarded as a strong predictor of risk for future PD (Ross et al., 2008). The most prominent olfactory impairment detected in the present model of PD pertains to the ability to recognize an odor as novel. This deficit is limited to the discrimination between odors belonging to the same category, i.e., non-social or social. In contrast, lesioned mice display intact discrimination between a non-social and a social odor, which are markedly different. A similar impairment has been observed in mice overexpressing human α-synuclein, which discriminate between strongly different scents, but lose gradually the ability to distinguish between citrus odors (Fleming et al., 2008). Analogous olfactory defects have also been reported in transgenic mouse models of dopamine dysfunction. For instance, a progressive age-dependent disruption of olfactory discrimination has been reported in mice with reduced expression of the vescicular monoamine transporter 2 (VMAT2) (Taylor et al., 2009), and in mice deficient for the dopamine transporter and the D2R (Tillerson et al., 2006). Overall, the decrease in olfactory discrimination observed in 6-OHDA-lesioned mice is in line with the anosmia described in parkinsonian patients, which is characterized by a variable intensity and often manifested as lack of ability to distinguish specific odors (Ward et al., 1983; Doty et al., 1988; Mesholam et al., 1998; Braak and Del Tredici, 2009).

Our data show that a bilateral injection of 6-OHDA in the striatum increases the immobility time in the forced swim test and in the tail suspension test, two standard behavioral paradigms indicative of depression. These findings are in line with previous work performed in the 6-OHDA-lesioned rat (Winter et al., 2007; Tadaiesky et al., 2008; Santiago et al., 2010; Drui et al., 2014); but see (Eskow Jaunarajs et al., 2012). Moreover, increased immobility in the forced swim and tail suspension tests have been described in MPTP-lesioned mice and in mice with reduced expression of VMAT2 (Mori et al., 2005; Fukui et al., 2007; Taylor et al., 2009). In contrast, Branchi et al. (2010) did not observe any difference in immobility time between sham and 6-OHDA-lesioned mice, in the forced swim test. One possible explanation for this discrepancy is that Branchi et al. utilized mice with a 43% depletion of striatal dopamine, which is lower than that reported in the present PD model (70%) (Bonito-Oliva et al., 2014). In addition, the two studies used different stereotaxic coordinates for the 6-OHDA injection, which may affect striatal territories distinctly innervated by substantia nigra and ventral tegmental area. For instance, in Branchi et al. striatal injections of 6-OHDA were performed at a more rostral and medial level in comparison to the present study, which targeted preferentially the dorsal-lateral striatum. It is possible that these latter coordinates led to a more pronounced depletion of dopamine in the substantia nigra. In this context, it should be noted that the loss of nigro-striatal dopaminergic neurons has been proposed to specifically mediate mood-related disorders in experimental parkinsonism (Drui et al., 2014).

The depressive-like phenotype observed in our PD mouse model was accompanied by increased thigmotaxis and reduced time spent in the open arms of the elevated plus maze, which are two standard behavioral parameters indicative of anxiety. These results are in agreement with the frequent co-morbidity between anxiety and depression observed in PD patients (Menza et al., 1993) and with a number of observations in experimental models. In the rat, lesion with 6-OHDA has been reported to exert anxiogenic-like effects (Tadaiesky et al., 2008; Chen et al., 2011); but see (Branchi et al., 2008). In the mouse, lesion with MPTP did not affect anxiety-related behavior (Vuckovic et al., 2008; Prediger et al., 2010) and striatal injection with 6-OHDA was even found to reduce anxiety in the elevated plus maze test (Branchi et al., 2010). The present results are more in line with work performed in VMAT2-depleted mice (Taylor et al., 2009) and indicate that the combined manifestation of depression and anxiety can be successfully modeled in a toxin-based mouse model of PD.

In this study we examined the ability of dopaminergic and noradrenergic drugs to counteract the depressive- and anxiety-like behaviors caused by the partial 6-OHDA lesion. The efficacy of L-DOPA in treating affective disorders associated to parkinsonism is currently debated, with data indicating that this therapy is ineffective (Marsh and Markham, 1973; Kim et al., 2009), or that it may even exacerbate some of these conditions (Damasio et al., 1971; Choi et al., 2000). This situation is reflected by similar inconclusive evidence from studies performed in animal models. Thus, sub-chronic administration of L-DOPA produced a partial improvement of the response of 6-OHDA-lesioned rats in a learned helplessness paradigm of depression (Winter et al., 2007), whereas chronic administration exacerbated the anxiogenic-like effect of the lesion (Eskow Jaunarajs et al., 2012). Our results indicate that repeated administration of L-DOPA does not reverse the depressive- and anxiety-like behaviors produced by partial bilateral lesion with 6-OHDA. In support of these results, studies in rat and non-human primate models of PD indicate that L-DOPA elicits an excessive efflux of dopamine from non-dopaminergic neurons in areas such as the prefrontal cortex and the hippocampus. This, in turn, could induce psychotic states, which would reduce potential beneficial effects and even exacerbate mood-related conditions (Eskow Jaunarajs et al., 2012; Engeln et al., 2014).

The lack of efficacy of L-DOPA prompted the analysis of alternative dopaminergic treatments. In particular, we focused on pramipexole, a D2R/D3R agonist, previously shown to reduce mood-related symptoms in parkinsonian patients (Leentjens et al., 2009; Barone et al., 2010). We found that treatment with pramipexole effectively reverted the increase in immobility time displayed by lesioned mice in the forced swim test, as well as the anxiogenic-like effect produced by 6-OHDA in the elevated plus maze test. These results indicate the reliability of the present model and its predictive validity to study effective treatments for psychiatric disorders associated to PD and refractory to standard L-DOPA therapy.

One important question concerns the contribution of dopamine and noradrenaline to the affective disorders observed in PD. Clinical evidence indicates that selective blockers of noradrenaline reuptake, such as reboxetine and desipramine, ameliorate mood-related symptoms (Lemke, 2002; Devos et al., 2008). It has also been proposed that noradrenergic fibers release dopamine in the cerebral cortex (Devoto et al., 2005, 2008), a finding which raises the question of the impact produced on dopamine transmission by the loss of noradrenaline innervation in brain regions primarily involved in depression and anxiety. The 6-OHDA lesion utilized in this study affects both dopamine and noradrenaline innervation (Bonito-Oliva et al., 2014). Therefore, to assess the contribution of these systems to mood-related conditions, we limited the lesion with 6-OHDA to the dopamine neurons by pre-treating the mice with the selective noradrenaline reuptake inhibitor, desipramine (Bonito-Oliva et al., 2014). Interestingly, preserving the noradrenaline innervation did not modify the depressive- and anxiogenic-like effects of 6-OHDA. Thus, in the present PD model, a partial loss of noradrenergic innervation, or a decrease of dopamine release from noradrenergic terminals in cortical regions, appear to have a negligible impact on affective dysfunctions. Notably, we also found that administration of reboxetine counteracts both depressive- and anxiety-like behaviors produced by the 6-OHDA lesion. In light of the aforementioned results, it is unlikely that these effects are exerted by promoting noradrenergic transmission. A more plausible explanation is that reboxetine acts by blocking dopamine uptake from noradrenaline terminals, which is particularly efficient in extra-striatal regions, including cerebral cortex and hippocampus (Moron et al., 2002; Borgkvist et al., 2012). Indeed, the partial effect exerted by 6-OHDA on TH neurons in the locus coeruleus (Bonito-Oliva et al., 2014), indicates that spared noradrenergic terminals may still be able to handle dopamine in forebrain target areas.

In conclusion, the present study shows that a variety of non-motor symptoms associated to PD can be reproduced in a mouse model generated by a partial bilateral lesion with 6-OHDA. In this model, subtle impairments of gait dynamics and reduced olfactory discrimination, reminiscent of early stage PD, are accompanied by depressive- and anxiety-like behaviors. These affective dysfunctions are refractory to treatment with L-DOPA, but reverted by the D2R/D3R agonist, pramipexole. Moreover, they are counteracted by reboxetine, an inhibitor of noradrenaline reuptake, but independent of the reduction of noradrenaline transmission produced by the 6-OHDA lesion. Thus, the anti-depressant and anxiolytic effects of reboxetine are most likely exerted via blockade of dopamine reuptake from noradrenergic fibers.

## Author contributions

Alessandra Bonito-Oliva: design of the study, statistical analysis and interpretation of data, drafting and revising of the manuscript; Débora Masini: design, statistical analysis and interpretation of gait and olfactory studies, revising of the manuscript; Gilberto Fisone: conception of the study, interpretation of data, revising of the manuscript.

### Conflict of interest statement

The authors declare that the research was conducted in the absence of any commercial or financial relationships that could be construed as a potential conflict of interest.
